# Four Cases of Albuminuria Complicating Pregnancy

**Published:** 1873-11

**Authors:** S. M. Bemiss

**Affiliations:** Professor of Theory and Practice of Medicine and Clinical Medicine, University of Louisiana


### Miscellaneous.

### Four Cases of Albuminuria Complicating Pregnancy.

By S. M. BEMISS, M. D., Professor of Theory and Practice of Medicine and Clinical Medicine, University of Louisiana.

[We omit in this extract the publication of cases I and II giving only cases III and IV., and the author's concluding remarks. ED.]

*Case III.* Was a lady of spare form and of a highly endowed nervous system, but with no history of family tendency to convulsions. She was delivered of her first child in the month of May, 1870. The labor was unusually rapid, for at the moment the accoucheur entered the room the fœtus was expelled, still enveloped with unbroken membranes. The bag was ripped open and a living child extracted. By the mother's reckoning, and the child's imperfect development and small size, the conclusion was well established that it was delivered a month before the normal period of utero-gestation.

On the 27th of March, 1872, her husband came to ask a prescription for a headache of which she was complaining. Some pills of blue mass, rhubarb and castile soap were ordered. This was about the beginning of the 35th week of her second pregnancy.

At 11 o'clock on the morning of the 28th she was seized with a very violent convulsion, followed in quick succession by others, the patient during the intervals remaining wholly unconscious.

At my request Dr. A. C. Holt was joined with me in the treatment of the case, and to his skillful assistance the successful result is in a due degree ascribable. The patient's feet were swollen, but there was no discernible puffiness of the countenance, we, however, felt assured the case was one of convulsions, associated with albuminous urine, and succeeded in getting a scruple dose of calomel into the stomach by placing it upon the tongue shortly after the first convulsion, and following it with spoonfuls of water. We determined to effect delivery as early as possible, and if necessary, as we then supposed it would be, to attempt forcible dilatation, turn the child and deliver by the feet. Preparatory to these measures a catheter was introduced into the bladder, and a sufficient quantity of urine brought away to enable us to determine that it was loaded with albumen.

Notwithstanding the patient's insensible state we were soon able to perceive by her automatic efforts at straining, and the coincident uterine movements, that labor pains were already inaugurated. These spontaneous efforts were greatly aided by a manual dilatation of the cervix, an early rupture of the membranes and especially, also, by a carefully regulated, but tolerably firm downward pressure exerted upon the fundus uteri, at each contraction. Delivery was accomplished by the middle of the afternoon. A small and feeble female child was the result, which yet survives, and is reported to be healthy. No difficulty attended the expulsion of the placenta, and the uterus was well contracted afterwards. The patient was brought under the influence of chloroform at the earliest practicable moment after the first convulsive seizure, and this influence was maintained during the whole time of labor. After delivery was effected the convulsions still continued to recur at intervals, seldom greater than thirty minutes, as they had done during the labor. The treatment pursued was, to give chloroform rapidly whenever muscular twitchings announced the approach of a convulsive seizure, and to suspend its use as soon as the convulsion had ceased. In this manner we were able to mitigate the violence of the seizures with a good deal of certainty, and quite frequently succeeded in preventing their occurrence. Happily, both for the physicians and the patient, however profound her insensibility was, fluids placed far back on the dorsum of the tongue were in part swallowed. This enabled us to medicate and nourish her We determined first to make trial of the efficacy of bromide potash in calming nervous excitability, and gave twenty grains every two hours. Three doses were given without benefit, after which it was omitted and twenty grains of hydrate chloral given, to be repeated as often as the patient's condition demanded. The chloral exercised a more obvious control over the convulsive seizures, and was repeated at intervals of from three to six hours. In the mean time the patient's bowels were well acted upon by the calomel and an enema of castor oil emulsion. The kidneys also appeared to be in active exercise of function, but as the evacuations were all emptied into the bed, it was not possible to determine with regard to urinary abnormities. During the 30th; the only changes in treatment were four enemata, each containing bromide potash ℨss. and tinct. digitalis ℨij; also by mouth four five-grain doses of valerianate of quinine. This medication took the place of the chloral, which was omitted. A blister 6x4 inches was placed on the back of the neck. The patient exhibited indications of very great nervous exhaustion, with a rapid, extremely feeble and irregular pulse, and on this account, as well as from the marked mitigation in violence and frequency of the spasms, the above changes were considered proper. For the same reasons the chloroform was almost wholly discontinued.

On the morning of the 31st the convulsions ceased, and did not again return. No precise reckoning was kept of the number of convulsions the patient endured, but it could scarcely have fallen short of fifty. The patient lay in a comatose state for more than a week, and did not become conscious of the fact of her delivery until one week after its occurrence. Indeed, for several months she continued to complain of loss of memory, and hesitated in conversation, with a vacant expression of face altogether unusual to her. The after treatment of this case consisted of iron, with small doses of nux vomica, liberal diet, and a sea shore residence. A careful examination of the urine two weeks after delivery proved it to be non-albuminous.

This patient has removed from this city, but I learn that she was very recently delivered of her third child without convulsions.

*Case IV.* This was a primipara of very diminutive figure and quite young, but up to the time of marriage suffering with no disease except painful menstruation. Her family history presented interesting points with regard to puerperal convulsions. Her mother died after giving birth to a second child, which was still-born. At the period of the mother's death the patient was old enough to remark that she had "dreadful convulsions and was very dropsical." A maternal aunt also died during childbirth, with precisely analogous symptoms.

Some months after her marriage, the patient had an abortion at about the sixth week. Within twelve months two others had ensued, at from the sixth to the eighth week. I was then asked to visit her, and found retroversion of the uterus. The uterus was without difficulty restored to a normal position, and recumbency with warm vaginal douches ordered. After a few days of this treatment, a Hodge's bar pessary was introduced and the patient set at liberty. Her menses were observed for the last time October 5—10, 1872.

I was requested to see her on the 26th of May, and found that she had been suddenly awakened in the latter part of the preceding night with pain in the head. This she described as being tensive, terrible and unbearable in character, obliging her to spring to her feet instantly. The subcutaneous areolar tissue was quite generally infiltrated. The feet and legs were very large, and pitted on pressure. The face had the peculiar waxy, anæmic look of Bright's disease. The conjunctiva were red, and when a cheek was pressed for a time upon her hand, or even the pillow, a crimson flush remained for a long period. There was no trouble with the sight. The patient was greatly annoyed with frightful dreams, but her intellection was perfect. The bowels had been a little more inactive than usual, but it had not been thought necessary to take anything to relieve them. The urine was scanty, and almost wholly coagulable by heat. Its specific gravity was 1.012. The pulse beat 64 to the minute. The patient's husband stated that within the three days prior to my visit she had increased in weight 5 lbs. For some day or two there had been anorexia. The child's movements were sufficiently vigorous; no other uterine phenomena. I ordered for her small doses of calomel and jalap, to be repeated every two hours; but as these were rejected, two doses of calomel, each five grains, were placed upon the tongue and washed down with spoonfulls of ice water. These were followed by bitartrate potash in cold water, and by comp. jalap powder. Vomiting frequently occurred, and very great quantities of a dark yellow and greenish fluid were thrown up. Notwithstanding the irritability of the stomach, the above named purgatives were urged upon the patient, for it appeared to me that the indication for hydragogue catharsis was too urgent to admit of any delay.

In the afternoon very free watery purging occurred, and was attended with complete relief to the pain in the head. The vomiting also ceased; the patient's complexion seemed more natural, and her whole condition was bettered. She was now left for the night, with instructions to take ℈j of chloral hydrate in the event that she suffered pain, or from sleeplessness.

May 27th. The pain came on during the night, about the same hour that it had occurred on the previous night. The chloral had not been given, the patient insisting that the cephalalgia was less severe, and that walking the floor would relieve it. It passed away in less than an hour, and at the time of my visit she was free from pain. The puffiness of the face was less marked, the urine very albuminous but increased in amount; specific gravity 1.015. She was ordered a teaspoonful three times daily of mur. tinct. iron; and simple syrup aa ʒiv, mix; at night xxx grs. of bromide potash, in camphor water ℥ij. The bromide was ordered as a substitute for the chloral, as the patient was found to have some prejudice against the latter. Bitartrate potash was dissolved in the water which she drank, in such amount as to keep up some hydragogue action of the bowels. The appetite being a little improved, milk, eggs, and the juices of a quickly cooked mutton chop were directed as food for the day.

May 28th. Headache again last night, secretion of urine still increasing; less albuminous; bowels free. Thinking that quinine might exercise some control over the headache, I ordered twelve grains in three doses; to continue the iron, and take the bromide potash at bed-time.

May 29th. Cephalalgic pain very violent during the latter part of the night. The quinine had been in part rejected. Urine scanty, loaded with albumen; specific gravity 1.011. Pulse 48 to the minute. Gave calomel grains five, to be followed in three hours by a powder containing ten grains of jalap and ten of bitartrate potash. This produced free catharsis with liquid stools, after which ℈j of bromide of quinine was dissolved in ʒiv of concentrated extract of coffee, by the addition of citric acid, and a teaspoonful ordered every fourth hour. At night fifteen grains of chloral were given, with orders to repeat the same quantity during the night, if restlessness or pain rendered it necessary. The iron was discontinued until otherwise directed.

May 30th. The patient passed a better night, having experienced a very slight return of the pain. After this, her improvement was uninterrupted. The urine steadily increased in quantity and specific gravity, until, on June 7th, it was 1.023; the deposit, after submitting it to heat, was scarcely one-tenth. Chloral was taken in doses of from 7½ to 15 grains nightly. The bowels were kept free by ptisans of bitartrate potash. The iron was taken twice daily, whenever the patient's stomach was in such a state as to enable her to take it without annoyance. At this date it was not thought necessary to continue my daily visits.

Previous to this date, however, I requested a consultation to decide in regard to the propriety of inducing labor for the safety of the child. The patient was very intelligent, and fully aware of the danger, either as it respected herself or offspring, and being intensely anxious to bear a living child, was willing to encounter an increase of hazard to herself to accomplish this end. She daily reported that the fœtal movements were becoming more feeble. Dr. A. C. Holt met me on the first of June. The result of the consultation was, that in view of the decided improvement in the maternal system it was better to trust the case further, in the hope that if the viability of the child had not already suffered irreparable injury, it might share in the benefits of the mother's improved nutrition. On the 13th of June the patient's urine was so abundant as to cause a fear on her part that some new form of disease was impending. It contained but little albumen, and had a specific gravity of 1.007. The patient's feet and legs were still very œdematous, and the pits from pressure persisted for a great while. Still her complexion was improved, and her appetite and digestion satisfactorily good.

One symptom was present which gave me considerable uneasiness. Her breasts were relaxed and pendulous, and on the under surface there were red patches, only in part effaceable by pressure. The superficial capillaries were the seat of a blood stasis which at many points had resulted in rupture and extravasation. This seemed to indicate a degree of systemic innutrition more profound than that ordinarily coincident with mere serious infiltration.

The patient was seized with light labor pains on the morning of June 22d, and was delivered in the early part of the evening without the least difficulty or complication, so far as it respects herself. The fœtus was dead, macerated, the cuticle separating wherever much pressure had been made during delivery. The bones of the head were moveable, and the cranial sac pouched to such an extent during delivery, and had such a fluctuating, fluid character of touch, that I believed hydrocephalic effusion had commenced prior to the child's death. The patient states that the last date of fœtal movement was Saturday, June 14th. This is now the 21st day after delivery, and the patient's recovery has progressed satisfactorily. The only anomaly, indeed, which has occurred, has been a violent pain in the right eye, followed by disturbance of vision. This has occurred twice, and in each instance followed rather prolonged use of the eyes in reading. These symptoms were temporary. I did not see her during their continuance, and at the periods of my visits nothing unusual could be observed in the appearance of the affected eye.

No examination has been made with the opthalmoscope.

### REMARKS.

These cases do not throw any additional light upon the controverted points of pathology of puerperal convulsions. All, except the third case, have occurred in small primiparous women, whose abdominal walls were unyielding. Consequently the doctrine, that pressure is the morbid starting point, is in the aggregate supported. But those who are willing to admit pressure as the pathologic factor, are divided as to the manner in which it works the mischief. The greater number hold that it is exerted upon the vascular system, and the albuminuria is the result of congestion, but Tyler Smith, Fordyce Barker, and others equally excellent as professional authorities, believe the injury is from nervous irritation. The symptoms in these cases all developed themselves in the latter half of pregnancy, while it is a known fact reflex disturbances in pregnancy belong peculiarly to the earlier months. I have no doubt that much of the disagreement in opinions regarding the pathology of puerperal convulsions is due to the fact that different causes may determine them. When different diseased states are grouped under one name, we become prone to consider the nomenclature and pathology as being alike unvarying. That they are not uncommonly due to the hysterical diathesis is undeniable. That the irritation of the uterine nerves may give rise to them in women who have never exhibited hysterical manifestations is extremely likely. All constitutions, under certain circumstances, are impressible to those causes which excite reflex disturbances. I have at this moment a lady under observation whom I have treated in quite a number of hysterical convulsions. She is now far advanced in the ninth month of pregnancy, and has had two very violent attacks of convulsions during this state. The urine has not a trace of albumen. But even if deprived of opportunity to make observations of the urine, or of a history of previous hysteria, we could confidently assert a diagnosis, basing it upon the condition of the patient in the intervals between the seizures. There is no coma. When she recovers from the exhaustion of her excessive muscular exertions, she is free from morbid manifestations. In another class, as those cases detailed in this paper, the convulsions are ascribable to some morbid process which is, with great uniformity, associated with albuminous urine. The most consistent explanation is, that the same pathological conditions which produce the albuminous urine, at the same time embarrass the renal functions, and hinder depuration of the blood. We must not, however, conclude that these two pathological states are inseparably connected. It is altogether probable that obstructed renal depuration may occasionally exist in sufficient degree to produce convulsions without albuminous urine, and the the latter very often occurs without convulsions. It is also not at all improbable that other derangements of the fluids of the system may give rise to convulsive seizures besides those due to any form of uremic poisoning. Indeed, Professor Brickell's suggestion, that "vitiations of the blood incident to pregnancy" which may in no wise depend on crippled kidney function, are yet sufficient to produce convulsions, seems altogether probable. But even in those cases, where the relation between the lesions of renal function and the convulsions is fully established, the gross pathology is very essentially different from that of Bright's disease. The uremic poisoning—the general tendency to serious infiltration, the liability to convulsive seizures and the demolition of blood constituency, show great parallelism. But putting out of view the chemical differences in the albumen in the two states of disease, one radical distinction rests upon the difficulty and tediousness of cure in Bright's disease, contrasted with the rapidity with which the albuminuria of pregnancy gets well when delivery eccurs. No doubt it sometimes happens that the puerperal state entails in some manner or other, permanent disease of the kidneys. Four years ago I attended a lady of this city who had suffered several abortions at very short intervals. After the last, she remained anæmic, complaining of unusual fatigue after physical exertion. She had light rigors, with loss of appetite, and a tendency to menstrual returns more frequently than in health. With these symptoms she had headache and was nervous. She was treated with quinine, chalybeates and cod liver oil. Very soon the debility increased so that she kept her bed. Her respiration became hurried, the heart's action was weak, and the pulsations irregular as it respects force. No morbid chest sounds could be detected. I thought the anomaly of the symptoms was properly explained by a strong hysterical tendency manifested in the early part of her case. There was no dropsical infiltration anywhere. No indications of uremia, unless the above named symptoms were such, and no statements on her part calculated to call attention to the urine. Nevertheless, desiring to use every means of acquainting myself with her case, I obtained a specimen of her urine and submitted it to chemical and microscopical examination. It contained in abundance albumen, casts, and blood corpuscles. In less than two days from this time, she died very suddenly, and without convulsions. No post-mortem.

I remember to have read with great interest, but have lost my references to it, a paper published in a French Medical Journal, giving an account of five cases of acute albuminuria occurring so shortly after abortions, as to afford indubitable evidence of the connection of cause and effect. It is impossible for me to aver, in the case just related, that the albuminuria may not have antedated the last abortion, which was only some six weeks before the patient's death. My belief, however, is, that it did not precede but followed it, and was determined in a great measure, if not wholly, by extension of the grave uterine lesions to the kidneys.

Whatever remarks upon treatment are made may be confined to the fourth case upon the record. A physician's successes, like those of a general, are commonly commended, even if shown to be in contravention to the rules either of science or common sense. In respect to the last recorded case, the treatment directed for the safety of the mother need scarcely be discussed. The indications for elimination—for hydragogues, were so pressing that the physician could make but little question concerning his measures of treatment. But it is not so irrelevant to enquire whether it might not have been better to have attempted something more than was done for the safety of the child.

The only possible step to be taken to save the child was the induction of premature labor. In the earlier progress of the case, I think there are two counts, which concurring, established the measure as unwarrantable. In the state of system the mother was in during the earlier stages of her illness, the danger of convulsions, and danger to her life therefrom were too imminent to justify induction of labor. The same considerations, touching the child were also to be weighed, in connection with the possible occurrence of convulsions.

The fierce, intolerable cephalalgia, and slow pulse, with which the patient suffered, are so generally the precursors of convulsions, that the physician incurs increased danger and resumes a grave responsibility if he attempts to incite labor in the face of such admonitions.

The arguments which led us to defer any procedures to induce labor, later in the progress of the case, have already been mentioned.—*New Orleans Med. and Surg. Journal.*

